# Periodontitis and gastrointestinal cancer: a nationwide cohort study of NHANES 2009–2014

**DOI:** 10.1186/s12889-025-21832-2

**Published:** 2025-02-27

**Authors:** Ke Pu, Ting Luo, Juan Li, Qian Tang, Yang Feng, Guodong Yang

**Affiliations:** 1 Department of Gastroenterology, Affiliated Hospital of North Sichuan Medical College, Nanchong, Sichuan China; 2 Statesboro Office, Southeast Medical Group, Atlanta, GA 30022 US; 3 Department of Neurosurgery, Xi’an NO.3 Hospital, the Affiliated Hospital of Northwest University, Xi’an, Shaanxi 710018 China; 4 Department of Gastroenterology, Beijing Shijitan Hospital, Capital Medical University, Beijing, 100038, China

**Keywords:** Gastrointestinal neoplasms, Periodontitis, Incidence, Mortality

## Abstract

The association between periodontitis and gastrointestinal tract (GIT) cancer has undergone extensive investigation. However, there is ongoing controversy regarding the impact of periodontitis on the incidence and mortality rates among GIT cancer patients. This study aims to clarify the relationship between periodontitis and the risk as well as mortality rates of GIT cancer.

Methods: The data utilized in this study were obtained from the National Health and Nutrition Examination Survey (NHANES) database for the years 2009–2014, which included a total of 10,706 participants from the United States. The incidence and mortality rates of GIT cancer were analyzed in relation to periodontitis. To ensure the reliability of the results, additional adjustments for covariates and stratification analyses were conducted.

Results: In the multivariate logistic regression analysis, various variables such as age, sex, race, BMI, income, education, smoking, alcohol consumption, hypertension, hyperlipidemia, and diabetes mellitus were adjusted for. The analysis revealed no positive association between periodontitis and an increased risk of total cancer or GIT cancer including colorectal cancer. However, periodontitis was found to be significantly associated with higher mortality rates among participants for all-cause (HR: 1.58; 95% CI: 1.26-2.00; *P* < 0.001), GIT cancer (HR: 1.65; 95% CI: 1.24–2.20; *P* < 0.001), and colorectal cancer (HR: 1.65; 95% CI: 1.24–2.19; *P* < 0.001) individually.

Conclusions: The study demonstrates that periodontitis is not associated with an increased risk of incidence for total cancer, or GIT cancer including colorectal cancer. However, it is significantly associated with a higher risk of mortality for all-cause, GIT cancer, and colorectal cancer among participants in the NHANES study.

**Supplementary Information:**

The online version contains supplementary material available at 10.1186/s12889-025-21832-2.

## Introduction

Periodontitis is a multifactorial chronic inflammatory disease characterized by the deposition of dental plaque and the progressive destruction of the tooth-supporting structures, including the periodontal membrane and alveolar bone. Common features include gingival inflammation and recession, attachment loss, deep probing depths, alveolar bone loss, mobility, bleeding on probing, and pathological tooth migration [1]. Periodontitis is one of the top ten most prevalent diseases worldwide, significantly burdening global health [2, 3]. Extensive research link periodontitis to systemic conditions, including cardiovascular disease, type 2 diabetes mellitus [4–6] and tumor progression due to systemic chronic inflammation [3, 5, 7]. Persistent inflammation in periodontitis promotes tumor growth through increased secretion of cytokines, chemokines, and angiogenic factors (8). Oral bacteria like *Porphyromonas gingivalis* and inflammatory responses, including macrophage activation and Th17 cell migration, further strengthen the link between periodontitis and tumors (9). Additional carcinogenic factors, such as human tumor viruses (e.g., Epstein Barr and herpes viruses) can induce immune suppression and bacterial overgrowth, leading to chronic inflammation and autoimmune responses. These process may cause DNA damage, cellular senescence, and oxidative stress, potentially contributing to tumor formation [6]. However, the mechanisms linking these conditions remain unclear.

Gastrointestinal tract (GIT) cancer is a leading cause of cancer incidence and mortality worldwide. According to the 2020 GLOBOCAN statistics, GIT cancers, including colon (6.0% of total cancer cases), liver (4.7%), stomach (5.6%), rectal (3.8%), and esophageal (3.1%) cancers, exhibit significantly high incidence rates, especially in Asian populations, followed by Europe and North America [8]. While early-stage treatment advances have improved survival, prognosis for advanced stages remains poor. Therefore, it is of utmost importance to investigate the susceptibility factors of digestive tract tumors for the purposes of prevention and understanding tumor development.

Currently, the association between periodontitis and GIT cancer has garnered significant attention among researchers. A growing body of evidence suggests that periodontitis is linked to increased incidence and mortality rates of esophageal, colorectal, and pancreatic cancers [9, 10]. However, the results of these studies remain controversial. One contributing factor to this controversy is the variation in the definition of periodontitis across different studies. Additionally, effectively controlling for common confounding factors, such as smoking, presents a challenge. Despite the potential role of periodontitis in cancer development, there is still lack of long-term cohort studies that comprehensively investigate its impact on the incidence and mortality rates of GIT cancer. In this study, we aim to address this controversy by examining the relationship between periodontitis and the occurrence of total cancer, GIT cancer, colorectal cancer, as well as related mortality rates. To achieve this, we conducted an investigation using the National Health and Nutrition Examination Survey (NHANES) database, which represents the US population. By utilizing this extensive dataset, we hope to validate our hypothesis that periodontitis increase the risk of total cancer, GIT cancer, and their associated mortality rates.

## Methods

### Study population

**Fig. 1:**
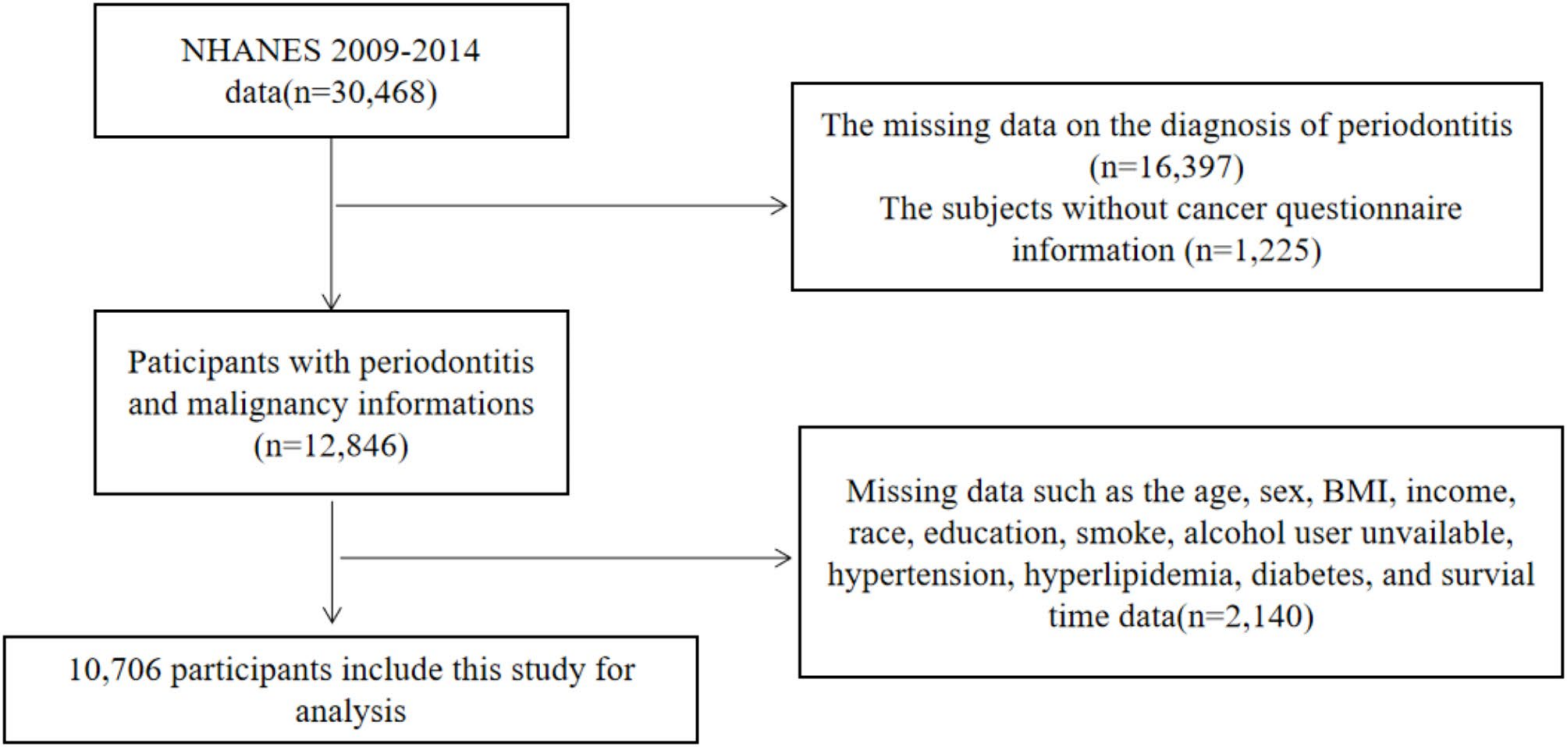
Flowchart of the participant selection process between periodontitis and cancers in adult participants of the NHANES Survey (2009–2014)

The study sample comprises three continuous cycles (2009–2010, 2011–2012 and 2013–2014) of the National Health and Nutrition Survey (NHANES), which aims to provide a representative sample of the US population. All data and materials are publicly available on the National Center for Health Statistics website (https://www.cdc.gov/nchs/nhanes/index.htm). The NHANES protocol was approved by the Institutional Review Board of the National Center for Health Statistics, Centers for Disease Control and Prevention, and all participants provided informed consent. A total of 30,468 participants aged 17 years or older were included in this study, 19,762 participants were excluded from the study due to incomplete data of periodontitis and cancers, and an additional 2,140 participants were excluded due to missing data on other covariates. Ultimately, 10,706 participants were included in the study (Fig. 1).

### Periodontitis

Clinical attachment level (AL) and probing pocket depth (PD) were evaluated by using periodontal probes to measure gingival recession and pocket depth at six sites on each tooth. Periodontitis was classified into mild periodontitis, moderate periodontitis and severe periodontitis according to the 2012 Centers for Disease Control and Prevention/American Academy of Periodontology case definitions (CDC/AAP) [11]. The classification of grade and severity of periodontitis is presented in Supplementary Table 1.

### Cancer

The medical conditions section of NHANES collects self-reported personal interview data on health condition [12]. Cancer diagnoses were determined through two questions in Medical Condition Questionnaire (MCQ): (1) “Have you ever been told by a doctor or other health professional that you had cancer or a malignancy of any kind?” (MCQ220); and (2) “What kind of cancer was it?” (MCQ220A, MCQ220B, and MCQ220C). After completing the online interview, trained interviewers collected and reviewed the questionnaires by virtue of the Computer-Assisted Personal Interviewing (CAPI) system [13].

### Mortality

The National Center for Health Statistics published Public-use Linked Mortality Files (LMF) for NHANES 1999–2014 [14]. These files contain information on mortality status and underlying causes of death. Cause-specific deaths were determined using the 10th Revision of the International Classification of Diseases (ICD-10) [15]. In this study, individuals with insufficient identifying data or those not available for public release were excluded.

### Covariates

Information on age, sex, race/ethnicity, education, poverty-income ratio (PIR), Body mass index (BMI), smoking status, alcohol, hypertension, hyperlipidemia, and diabetes mellitus in NHANES were collected through self-administered questionnaires and laboratory examinations. Race and ethnicity were classified into categories such as Non-Hispanic White, Non-Hispanic Black, Mexican American, and Other Race. Education was categorized as less than high school, high school graduate or equivalent, some college or associate degree, and college graduate or above. PIR was divided into two categories with a cutoff of 1, with a value of less than one indicating that the household is below the poverty line. Trained health technicians at the mobile examination center measured height (to the nearest 0.1 cm) and body weight (to the nearest 0.1 kg). BMI was calculated as weight (kg) divided by height squared (m²). Smoking status was categorized as never smokers (smoked less than 100 cigarettes in their lifetime), former smokers (smoked more than 100 cigarettes but not at the time of the survey), and current smokers (smoked more than 100 cigarettes in their lifetime and currently smoke). Alcohol intake was classified as none (0 drink/d), former drinking, and current drinking. Hypertension was defined as follows: (1) average systolic blood pressure ≥ 140 mmHg, (2) average diastolic blood pressure ≥ 90 mmHg, (3) self-reported hypertension, (4) individuals taking prescribed antihypertensive medications [16]. Hyperlipidemia was determined based on criteria such as total cholesterol ≥ 200 mg/dL, triglycerides ≥ 150 mg/dL, low-density lipoprotein ≥ 130 mg/dL, and high-density lipoprotein < 40 mg/dL in males or < 50 mg/dL in females [17]. Diabetes mellitus was defined as follows: (1) self-reported diabetes mellitus, (2) fasting plasma glucose level of at least 7 mmol/L, two-hour oral glucose tolerance test of at least 11.1 mmol/L, or hemoglobin A1c (HbA1c) level of at least 6.5%, and (3) use of oral glucose-lowering medications or insulin [18].

### Statistical analysis

The analyses in the study accounted for the complex survey design of NHANES utilizing appropriate subsample weights, strata, and primary sampling units as recommended by NHANES protocol. Population characteristics were summarized using means and standard errors for continuous variables, and percentages and standard errors for categorical variables. Differences between groups were assessed using the Wilcoxon rank-sum test for continuous variables and the chi-squared test for complex survey samples for categorical variables. Logistic regression models were used to assess the odds ratios (ORs) of individuals with periodontitis for overall cancer, GIT cancer, and colorectal cancer. Cox regression models were employed to calculate hazard ratios (HRs) with 95% confidence intervals (CIs) for all-cause mortality, totalcancer mortality, and GIT cancer mortality among individuals with periodontitis. All regression models were adjusted for potential confounders. The initial models included adjustments for age, sex, race, BMI, income, and education, while the second models additionally adjusted for smoking, alcohol consumption, hypertension, hyperlipidemia, diabetes mellitus, and NHANES survey cycles. The study examined also explored the potential effect modifications by sex on the association between periodontitis and the incidence and mortality of total cancer, GIT cancer, and colorectal cancer through stratification analysis. Kaplan-Meier curves were used to visualize the rates of all-cause mortality, total cancer mortality, GIT cancer-related mortality, and colorectal cancer mortality. The statistical analyses were performed using R (version 4.0.2, www.R-project.org).

## Results

From the NHANES 2009–2014 data, a total of 10,706 participants were included in the study, all of them completedboth periodontal disease questionnaire surveys and tumor surveys. Table 1 provides a summary of the baseline demographic data and health-related characteristics of the participants, including the distribution of those associated with periodontitis within theoverall study population. The mean age of the participants was 50.84 years, with a standard deviation (SD) of 0.24 years. The poverty-income mean ratio of 3.15 with an SD of 0.05. Males accounted for 49.22% of the cohort. Among the participants, 18.77% were current smokers, and 68.76% were current alcohol consumers. The prevalence of hypertension, hyperlipidemia and diabetes mellitus were 43.93%, 72.91%, and 13.64%. Regarding tumors diagnosis, 9.43% of the participants had tumors, with 0.71% having digestive tract tumors and 0.64% having colorectal cancer. Significant differences (*p* < 0.05) were observed between participants with periodontitis and those with periodontal health in various health-related variables, including age, poverty-income ratio, gender, race, education, BMI, smoking status, alcohol consumption, hypertension, hyperlipidemia, and diabetes mellitus.

**Table 1:** Survey-weighted characteristics of the study population by periodontitis status

Characteristic		Non-periodontitis	Periodontitis	*P*-value
	Total	None (*n* = 5218)	Periodontitis (*n* = 5488)	
Age	50.84 ± 0.24	48.09 ± 0.28	54.59 ± 0.34	< 0.001
Poverty-income ratio	3.15 ± 0.05	3.50 ± 0.05	2.68 ± 0.05	< 0.001
Male	5270(49.22)	2083(42.01)	3187(58.06)	< 0.001
Race				< 0.001
Mexican American	1523(14.23)	550( 5.63)	973(11.44)	
Non-Hispanic Black	2229(20.82)	863( 8.00)	1366(14.41)	
Non-Hispanic White	4585(42.83)	2611(74.62)	1974(59.85)	
Others	2369(22.13)	1194(11.75)	1175(14.30)	
Education				< 0.001
≤ High school	4805(44.93)	1702(26.42)	3103(49.50)	
> High school	5889(55.07)	3512(73.58)	2377(50.50)	
BMI				0.002
Underweight/normal	2861(26.89)	1465(28.25)	1396(25.05)	
Overweight	3674(34.53)	1780(35.62)	1894(35.36)	
Obesity	4106(38.59)	1947(36.13)	2159(39.58)	
Smoking				< 0.001
Never	6017(56.22)	3376(64.12)	2641(46.04)	
Former	2676(25)	1187(24.57)	1489(28.36)	
Current	2009(18.77)	655(11.31)	1354(25.59)	
Alcohol				< 0.001
Never	1350(13.68)	625( 9.96)	725(10.91)	
Former	1734(17.56)	660(11.71)	1074(19.04)	
Current	6788(68.76)	3535(78.33)	3253(70.05)	
Hypertension	4703(43.93)	1901(34.51)	2802(47.88)	< 0.001
Hyperlipidemia	7804(72.91)	3696(71.73)	4108(75.51)	0.002
Diabetes mellitus	1451(13.64)	481( 7.44)	970(14.78)	< 0.001
Total cancer	1010(9.43)	447(10.05)	563(11.66)	0.07
Gastrointestinal tract caner	69(0.71)	25(0.59)	44(0.79)	0.34
Colorectal cancer	62(0.64)	23(0.57)	39(0.74)	0.43

Values are means ± SD for continuous variables. p Value was calculated by weighted linear regression model for continuous variables and weighted chi-square test was performed for categorical variables. a Unweighted frequency counts and weighted percentages are shown

Abbreviations: BMI, Body mass index

**Fig. 2:**
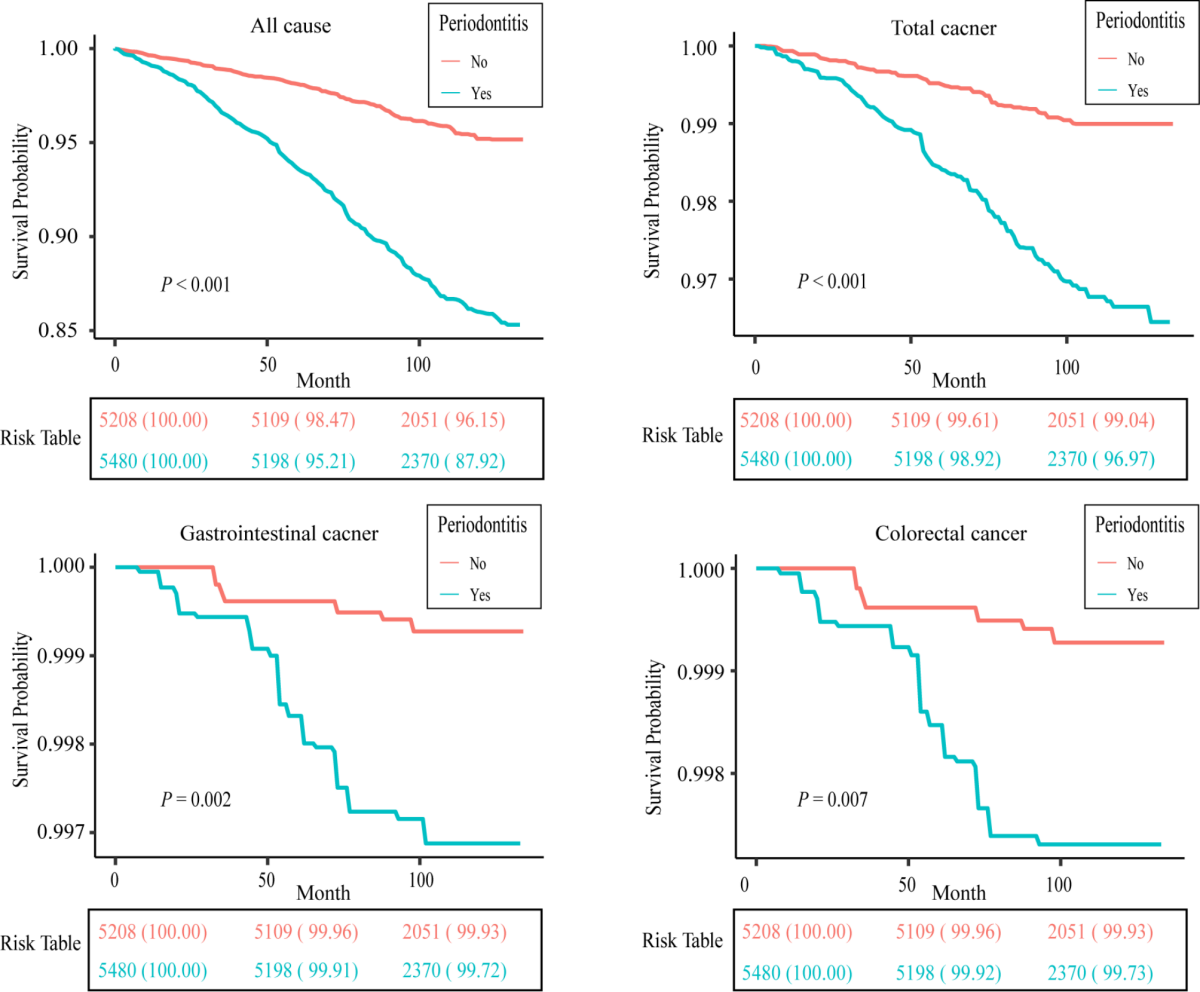
The Kaplan-Meier survival curves to analyze the impact of periodontitis and periodontal health on the occurrence of all-cause death, total cancer, gastrointestinal cancer, and colorectal cancer over a follow-up period of 100 months

In the multivariate analysis, it was found that there was a positive association between periodontitis and an increased risk of tumors in Model 1 (OR: 1.25, 95% CI: 1.02–1.55, *P* = 0.04). However, after adjusting for all explanatory variables in Model 2, this association did not persist (OR: 1.16, 95% CI: 0.94–1.44, *P* = 0.16). Similarly, no positive risk association was observed between periodontitis and GIT cancer (OR: 1.06, 95% CI: 0.52–2.15, *P* = 0.87) or colorectal cancer (OR: 1.04, 95% CI: 0.52–2.07, *P* = 0.90) (Table 2). Furthermore, subgroup analysis based on sex indicated that both male and female individuals with periodontitis were not associated with an increased risk of total cancer, GIT cancer, and colorectal cancer (Table 3). Regarding mortality rates, our finding revealed that participants with periodontitis had significantly higher rates of all-cause mortality (*P* < 0.001), total cancer mortality (*P* < 0.001), GIT cancer mortality (*P* = 0.009), and colorectal cancer mortality (*P* = 0.02) compared to the non-periodontitis group (Table 4). Kaplan-Meier survival analysis, depicted in Fig. 2, showed that subjects with periodontitis had significantly shorter survival times than subjects without periodontitis in all four groups (*P* < 0.0001). This suggests that periodontitis may increase the risk of death related to all-cause, all-cancer, GIT cancer including colorectal cancer.

**Table 2:** Risk analysis of total cancer, gastrointestinal tract cancer and colorectal cancer relative to periodontal status in the survey-weighted multivariable-adjusted logistic regressions

Variable	NO.of subjects	NO.of cancers	Unadjusted model		Age, sex-adjusted model		Model1		Model2	
			OR (95%CI)	*P*- value	OR (95%CI)	*P*- value	OR (95%CI)	*P*- value	OR (95%CI)	*P*- value
Total cancer										
No	5,218	447	1.00 (ref)		1.00 (ref)		1.00 (ref)		1.00 (ref)	
Periodontitis	5,488	563	1.18 (0.98,1.42)	0.07	1.03 (0.86,1.25)	0.70	1.25 (1.02,1.55)	**0.04**	1.16 (0.94,1.44)	0.16
Gastrointestinal tract cancer										
No	5,218	25	1.00 (ref)		1.00 (ref)		1.00 (ref)		1.00 (ref)	
Periodontitis	5,488	44	1.29 (0.67,2.49)	0.44	1.11 (0.58,2.12)	0.75	1.23 (0.61, 2.50)	0.55	1.06 (0.52,2.15)	0.87
Colorectal cancer										
No	5,218	23	1.00 (ref)		1.00 (ref)		1.00 (ref)		1.00 (ref)	
Periodontitis	5,488	39	1.27 (0.66,2.44)	0.50	1.11 (0.58,2.10)	0.80	1.19 (0.59, 2.39)	0.60	1.04 (0.52,2.07)	0.90

Model 1: adjustments for age, sex, race, BMI, income, and education

Model 2: initial model with additional adjustments for smoking, alcohol, hypertension, hyperlipidemia and diabetes, and NHANES survey cycles

Abbreviations: CI, confidence interval; OR, odds ratio

**Table 3:** Sex specific adjusted odds ratios (ORs) (95%CI) for total cancer, gastrointestinal tract cancer, and colorectal cancer risk relative to periodontal status in the survey-weighted multivariable-adjusted logistic regressions

Variable	Unadjusted model		Age, sex adjusted model		Model1		Model2	
	OR (95%CI)	*P*- value	OR (95%CI)	*P*- value	OR (95%CI)	*P*- value	OR (95%CI)	*P*- value
Total cancer								
Male	1.23(0.90,1.68)	0.19	1.00(0.72, 1.39)	0.99	1.31(0.92, 1.87)	0.13	1.22(0.85,1.75)	0.27
Female	1.25(0.99,1.58)	0.06	1.06(0.85,1.34)	0.59	1.20(0.93,1.55)	0.16	1.11(0.85,1.45)	0.43
Gastrointestinal tract cancer								
Male	1.02(0.37,2.84)	0.97	0.81(0.29, 2.25)	0.67	0.92(0.33, 2.57)	0.86	0.73(0.31, 1.69)	0.45
Female	1.77(0.68,4.65)	0.24	1.44(0.55, 3.79)	0.45	1.39(0.48, 4.02)	0.53	1.22(0.41, 3.67)	0.72
Colorectal cancer								
Male	0.91(0.32,2.54)	0.85	0.72(0.25,2.01)	0.52	0.90(0.30, 2.65)	0.84	0.72(0.30, 1.75)	0.46
Female	1.77(0.67,4.72)	0.24	1.44(0.54, 3.85)	0.46	1.42(0.48, 4.18)	0.51	1.26(0.42, 3.81)	0.67

Model 1: adjustments for age, sex, race, BMI, income, and education

Model 2: initial model with additional adjustments for smoking, alcohol, hypertension, hyperlipidemia and diabetes, and NHANES survey cycles

Abbreviations: CI, confidence interval; OR, odds ratio

**Table 4:** All-cause mortality, total cancer, gastrointestinal tract cancer, and colorectal cancer mortality relative to periodontal status among study subjects in NHANES 2009–2014

Variable (*N*, %)	Total	Non-periodontitis	Periodontitis	*p*-value
	(*n* = 10,688)	(*n* = 5208)	(*n* = 5480)	
All-cause	919(8.60)	240( 4.61)	679(12.56)	< 0.001
Total cancer	223(2.08)	59( 1.13)	164(2.99)	< 0.001
Gastrointestinal tract cancer	23(0.22)	5(0.10)	18(0.33)	0.009
Colorectal cancer	21(0.21)	5(0.10)	16(0.30)	0.02

To explore the association between periodontitis and mortality rates for all-cause, total cancer, GIT cancer, and colorectal cancer, multivariable Cox regression models applied. As shown in Table 5, subjects with periodontitis had more higher all-cause mortality (HR:3.26, 95% CI: 2.67–3.98, *P* < 0.001), total cancer mortality (HR: 3.37, 95% CI: 2.25–5.03, *P* < 0.001), GIT cancer mortality (HR: 3.32, 95% CI: 2.64–4.18, *P* < 0.001), and colorectal cancer mortality (HR: 3.32, 95% CI: 2.64–4.18, *P* < 0.001) than those without periodontitis in the unadjusted model. However, when controlling for all confounding factors in model 2, except for the total cancer mortality, periodontitis remained significantly associated with increased all-cause mortality, GIT cancer mortality, and colorectal cancer mortality (*p* < 0.001). Furthermore, the mortality risk of periodontitis for all-cause, total cancer, GIT cancer, and colorectal cancer was analyzed in a sex-stratified subgroup (Table 6). In both the male and female groups, periodontitis was significantly associated with higher HRs for all-cause mortality, total cancer mortality, GIT cancer mortality, and colorectal cancer mortality in the unadjusted models (*P* < 0.001). After adjusting for all included variables using Model 2, periodontitis remained significantly associated with the mortality rate of all-cause, GIT cancer, and colorectal cancer (*P* < 0.05), but it was not significantly associated with total cancer mortality.

**Table 5:** Risk of all-cause, total cancer, gastrointestinal tract cancer, and colorectal cancer mortality in NHANES 2009–2013 among study subjects with periodontitis

Variable	Unadjusted model		Age, sex-adjusted model		Multivariate model1		Multivariate model2	
	HR (95%CI)	*P*-value	HR (95%CI)	*P*-value	HR (95%CI)	*P*-value	HR (95%CI)	*P*-value
All cause mortality (*n* = 919)								
No	1.00 (ref)		1.00 (ref)		1.00 (ref)		1.00 (ref)	
Periodontitis	3.26 (2.67,3.98)	< 0.001	1.86 (1.50,2.30)	< 0.001	1.70 (1.34,2.14)	< 0.001	1.58 (1.26,2.00)	< 0.001
All cancer mortality (223)								
No	1.00 (ref)		1.00 (ref)		1.00 (ref)		1.00 (ref)	
Periodontitis	3.37 (2.25,5.03)	< 0.001	1.44 (0.97,2.14)	0.07	1.47 (0.98,2.22)	0.07	1.30 (0.91,1.85)	0.15
Gastrointestinal tract cancer (23)								
No	1.00 (ref)		1.00 (ref)		1.00 (ref)		1.00 (ref)	
Periodontitis	3.32 (2.64,4.18)	< 0.001	1.94 (1.54,2.46)	< 0.001	1.77 (1.35,2.32)	< 0.001	1.65 (1.24,2.20)	< 0.001
Colorectal cancer (21)								
No	1.00 (ref)		1.00 (ref)		1.00 (ref)		1.00 (ref)	
Periodontitis	3.31 (2.64,4.17)	< 0.001	1.94 (1.53,2.45)	< 0.001	1.77 (1.35,2.32)	< 0.001	1.65 (1.24,2.19)	< 0.001

Model 1: adjustments for age, sex, race, BMI, income, and education

Model 2: initial model with additional adjustments for smoking, alcohol, hypertension, hyperlipidemia and diabetes, and NHANES survey cycles

Abbreviations: CI, confidence interval; HR, Hazard ratio

**Table 6:** Sex specific risk analysis of all-cause, total cancer, gastrointestinal tract cancer, and colorectal cancer mortality in NHANES 2009–2013 among study subjects with periodontitis

Variable	NO.of subjects *n* = 10,688	NO.of events	Event rate (%)	Unadjusted model		Age, sex-adjusted model		Model1		Model2	
				HR (95%CI)	*P*- value	HR (95%CI)	*P*- value	HR (95%CI)	*P*- value	HR (95%CI)	*P*- value
All cause (919)											
Male	5,428	534	4.61	3.10 (2.42,3.96)	< 0.001	1.92 (1.47,2.49)	< 0.001	1.74 (1.28,2.37)	< 0.001	1.63 (1.17,2.27)	0.004
Female	5,260	385	12.39	3.33 (2.54,4.36)	< 0.001	1.78 (1.36,2.33)	< 0.001	1.63 (1.20,2.23)	0.002	1.52 (1.10,2.09)	0.01
Total cancer (223)											
Male	5,428	134	0.48	3.27 (1.94,5.52)	< 0.001	1.53 (0.90,2.61)	0.12	1.70 (0.97,2.99)	0.07	1.53 (0.92,2.54)	0.10
Female	5,260	89	0.80	3.39 (2.16,5.31)	< 0.001	1.35 (0.87,2.10)	0.18	1.24 (0.77,1.99)	0.37	1.05 (0.66,1.66)	0.85
Gastrointestinal tract cancer (23)											
Male	5,428	14	0.10	3.18 (2.37,4.27)	< 0.001	4.02 (0.84,19.15)	0.08	1.80 (1.24,2.62)	**0.002**	1.66 (1.08,2.54)	0.02
Female	5,260	9	0.33	3.41 (2.44,4.75)	< 0.001	1.46 (0.31,6.79)	0.63	1.73 (1.17,2.55)	0.01	1.63 (1.10,2.40)	0.01
Colorectal cancer (21)											
Male	5,428	12	0.10	3.17 (2.37,4.24)	< 0.001	2.02 (1.47,2.79)	< 0.001	1.80 (1.24,2.62)	0.02	1.65 (1.08,2.54)	0.02
Female	5,260	9	0.30	3.41 (2.44,4.75)	< 0.001	1.85 (1.34,2.54)	< 0.001	1.73 (1.17,2.55)	0.01	1.63 (1.10,2.40)	0.01

Abbreviations: CI, confidence interval; HR, Hazard ratio

## Discussion

In this study, a total of 10,706 participants from the NHANES 2009–2014 database were included, within three survey circles. After adjusting for confounding factors, the primary finding of the research indicates that periodontitis is not associated with an increased risk of total cancer, GIT cancer, and colorectal cancer. However, subjects with periodontitis exhibited shorter survival times and higher mortality rates for all-cause, GIT cancer, and colorectal cancer compared to those with periodontal health.

Previous epidemiologic studies investigating the association between periodontitis and the incidence risk of various cancers have produced inconsistent results. For instance, a cohort study conducted in Taiwan found that periodontitis was not associated with an increased risk of total cancer, excluding oral cancer [19]. Conversely, a cohort study by Michaud DS et al. [20] from the Health Professionals’ Follow-up Study (HPFS) reported that individuals with advanced periodontitis had a 2.5-fold increased risk of total cancer compared to those without periodontitis. Later, Michaud DS et al. [10] updated their findings using data from the Atherosclerosis Risk in Communities study (ARIC) and observed that severe periodontitis was associated with an increased risk of total cancer compared to individuals with no or mild periodontitis. However, inconsistencies remain in the findings of Michaud DS et al. regarding colorectal cancer in the two previously mentioned studies. While no association was found between periodontitis and colorectal cancer in HPFS cohort population [20], severe periodontitis was linked to elevated risk for colorectal cancer among never-smokers [10]. Moreover, a meta-analysis have demonstrated a significant association between periodontitis and an increased risk of total cancer, particularly Gastrointestinal tract (GIT) cancer other than colorectal cancer [21]. Similarly, another study identified periodontitis as a risk factor for GIT cancer [22]. Other studies did not support periodontitis as a risk factor for total colorectal cancer but identified it as being associated with an increased risk specifically for proximal colorectal cancer [23, 24]. Overall, the relationship between periodontitis and the risk of total cancer, GIT cancer, and colorectal cancer remains a subject of ongoing debate and is yet to be conclusively established.

Our study revealed that periodontitis is associated with shorter survival time and a higher mortality rate for all-cause cancer, GIT cancer, and colorectal cancer compared to individuals with healthy periodontal conditions. A meta-analysis conducted in 2020, which included 48 cohorts and 5.71 million participants, indicated that periodontitis is associated with an increased risk of all-cause mortality [25]. Subsequent cohort studies also confirmed similar conclusions [26, 27]. However, the findings of our study regarding the association between periodontitis and total cancer mortality are inconsistent with previous research. A meta-analysis demonstrated a positive relationship between periodontal disease and total cancer mortality [28]. This discrepancy may be explained by differences in the cancer spectrum observed in the NHANES cohort population compared to other cohorts, leading to lower number of periodontitis-related tumors and uncertain results. Additionally, the reliance on self-reported data, which could introduce misclassification of periodontal disease, may have affect the observed associations compared to studies utilizing clinical examinations. Although our study did not find an association between periodontitis and a higher mortality risk for total cancer, we did observe periodontitis-related mortality for GIT cancer and colorectal cancer. The GIT cancer subgroup includes various cancers of the gastrointestinal tract, but due to the limited number of cases, we focused specifically on colorectal cancer. Currently, there are fewer studies exploring the association between periodontitis and GIT cancer as well as colorectal cancer. Sung CE et al. [27] demonstrated a significant association between periodontitis and a higher mortality risk for GIT and colorectal cancer in subjects with *H. pylori* infection. Another meta-analysis indicated that periodontitis is associated with increased mortality in GIT cancer, but not with colorectal cancer mortality [22]. Further research, including longer follow-up periods, multi-center trials, and multinational studies, may be required to support these findings.

The relationship between periodontitis and gastrointestinal cancer remains incompletely understood. However, studies have demonstrated that oral microbiota can migrate and colonize the intestines, particularly in immunocompromised individuals, where a higher prevalence of pathogenic bacteria exists [29]. In colorectal cancer tumor tissue, Komiya et al. [30] identified *Fusobacterium nucleatum* (*F. nucleatum*) originating from the oral cavity, suggesting the translocation of oral microbiota. Genomic research further supports the migration of oral microbiota to other organs within the digestive system [31]. Oral bacteria can enter the bloodstream through normal dental activities and dental surgeries, potentially causing cause oral physical damage [32, 33]. Experimental models of periodontitis have shown that oral inflammation plays a crucial role in the systemic colonization of oral bacteria through dissemination in the circulation [34]. Enteral transmission is another route for oral bacteria. Normally, ingested oral bacteria rarely penetrate and colonize the healthy gut mucosa due to the immune defense system of the GIT. However, the immune status of the host and other contributing factors such as antibiotic misuse and an unhealthy diet can influence the translocation and colonization of oral bacteria [32]. Once oral microbiota successfully translocate and colonize the intestines, the compromised intestinal barrier can induce chronic inflammation, activate abnormal immune responses, and trigger carcinogenic signaling pathways. These factors potentially impact the development and progression of tumors in the digestive system. Several studies on *F. nucleatum* have demonstrated its ability to promote the production of pro-inflammatory cytokines and chemokines, creating an inflammatory microenvironment conducive to tumor growth [35]. *F. nucleatum* can also modulate macrophage polarization states to facilitate colorectal cancer metastasis and assist tumor cells in evading killing by NK cells through its interaction with Fap2 [36, 37]. Additionally, *F. nucleatum* can activate carcinogenic signaling pathways by interacting with E-cadherin on colorectal cancer cells, thereby promoting tumor proliferation [38]. Similarly, *Porphyromonas gingivalis* (*P. gingivalis*), a key pathogenic bacterium in periodontitis, induces inflammation, anti-apoptotic effects, and suppression of immune cells. *P. gingivalis* activates the Nuclear factor-kappa B (NF-κB) pathway, leading to the overexpression of tumorigenic cytokines. Moreover, it can induce the epithelial-mesenchymal transition, promoting tumor metastasis by upregulating ZEB1 and MMPs. *P. gingivalis* also exerts an anti-apoptotic effect through ATP-dependent and PI3K/Akt signaling pathways. Furthermore, *P. gingivalis* induces the expression of programmed PD-L1, which inhibits the function of effector T cells, enabling tumor cells to evade immune responses [39]. However, further research is essential to gain a comprehensive understanding of the intricate interactions between oral microbiota and digestive system tumors.

This study has several limitations. *Firstly*, the use of self-reported periodontitis based on participant-filled questionnaires introduces a potential bias. The NHANES database did not update data for participants who developed periodontal disease during the follow-up period, possibly including them in the non-periodontal disease group. It is unknown whether tumor-related incidence and mortality were included when the periodontitis accepted treatment. *Secondly*, our study did not investigate the relationship between the severity of periodontitis and cancers, as this risk trends was not found to be influenced by the severity of periodontitis in our dataset. Additionally, as a cross-sectional study, the causal relationship between periodontitis and cancer incidence or mortality still be unknown. *Thirdly*, the NHANES database also lacked tumor clinicopathological information, leaving the association between periodontal disease and the clinicopathological features of tumors unknown. Due to the limited number of cases of gastrointestinal malignancies, we were unable to analyze the relationship between periodontitis and other digestive cancers individually. *Finally*, other confounding factors, such as the frequency of flossing and the number of visits to the dentist, could have potentially influenced the study results. Nevertheless, this study has notable strengths. It utilized the nationally representative NHANES database, which is linked to mortality data. The data collection process was rigorous, and the study employed weighted analysis, enhancing the reliability of the results and enabling generalizability to the population level. Furthermore, the study accounted for common and extensive confounding factors through adjustment in the analysis. Additionally, this study investigated the relationship between the incidence and mortality rates of tumors in different genders of patients with periodontal disease, an aspect that has been rarely explored in previous reports.

In conclusion, a significant correlation has been found between periodontitis and decreased survival outcomes in patients with gastrointestinal cancers, specifically colorectal cancer. This association persists even after adjusting for health-related variables, highlighting the substantial impact of periodontitis on the mortality from all causes, gastrointestinal cancers, and colorectal cancer. Although the precise underlying mechanisms for this association are not fully understood, further research is necessary to elucidate them and ultimately enhance long-term survival rates for individuals with cancer.

## Electronic supplementary material

Below is the link to the electronic supplementary material.

Supplementary Material 1

## Data Availability

No datasets were generated or analysed during the current study.
