# Supplementary material for: Periodontitis and gastrointestinal cancer: a nationwide cohort study of NHANES 2009–2014
Source: BMC Public Health. 2025 Feb 27;25:804. doi: 10.1186/s12889-025-21832-2 (PMC11869642; doi:10.1186/s12889-025-21832-2)
Supplement: Supplementary file 1 — Supplementary Material 1 [file 12889_2025_21832_MOESM1_ESM.docx]

**Periodontitis and gastrointestinal cancer: A nationwide cohort study of NHANES 2009-2014**

Supplementary table 1 Definitions of subjects according to different severity of periodontitis

| Subject Definition | Subject Definition |
| --- | --- |
| No periodontitis | No evidence of mild, moderate, and severe periodontitis |
| Mild periodontitis | ≥two interproximal sites with attachment loss (AL) ≥3 mm and <4mm  and ≥two interproximal sites with probing depth (PD) ≥4 mm not on the same tooth, or one site with PD ≥5 mm |
| Moderate periodontitis | ≥two interproximal sites with AL≥4 mm and <6mm not on the same tooth or  ≥two interproximal sites with probing depth (PD) ≥5 mm not on the same tooth |
| Severe periodontitis | ≥two interproximal sites with AL ≥6 mm not on the same tooth and ≥one or more interproximal sites with PD ≥5 mm |
